# Novel Aryl Phosphate for Improving Fire Safety and Mechanical Properties of Epoxy Resins

**DOI:** 10.3390/polym16213049

**Published:** 2024-10-30

**Authors:** Yue Xu, Wenjia Zhang, Ru Yin, Jun Sun, Bin Li, Lubin Liu

**Affiliations:** 1 Heilongjiang Key Laboratory of Molecular Design and Preparation of Flame Retarded Materials, College of Chemistry, Chemical Engineering and Resource Utilization, Northeast Forestry University, Harbin 150040, China; xuyue752758540@163.com (Y.X.); wenjiazhang2021@163.com (W.Z.); yu18814514176@163.com (R.Y.); libinzh@163.com (B.L.); 2 National and Local Joint Engineering Laboratory for Ecological Utilization of Biological Resources, Northeast Forestry University, Harbin 150040, China; 3 State Key Laboratory of Organic-Inorganic Composites, Beijing University of Chemical Technology, Beijing 100029, China

**Keywords:** aryl phosphate, epoxy resins, flame retardancy, mechanical performance, flame retardant mechanism

## Abstract

Epoxy resins (EPs) are highly flammable, and traditional flame retardant modifications often lead to a significant reduction in their mechanical performance, limiting their applications in aerospace and electrical and electronic fields. In this study, a novel flame retardant, bis(4-(((diphenylphosphoryl)oxy)methyl)phenyl)phenyl phosphate (DMP), was successfully prepared and introduced into the EP matrix. When the addition of DMP was 9 wt%, the EP/9 wt% DMP thermosets passed the UL-94 V-0 rating, and their LOI was increased from 24.5% of EP to 35.0%. With the introduction of DMP, the phosphoric acid compounds from the decomposition of DMP promoted the dehydration and charring of the EP matrix, and the compact, dense char layer effectively exerted the shielding effect in the condensed phase. Meanwhile, the produced phosphorus-containing radicals played a quenching effect in the gas phase. As a result, the peak heat release rate (PHRR) and total heat release (THR) of EP/9 wt% DMP were reduced by 68.9% and 18.1% compared to pure EP. In addition, the polyaromatic structure of DMP had good compatibility with the EP matrix, and the tensile strength, flexural strength and impact strength of EP/9 wt% DMP were enhanced by 116.38%, 17.84% and 59.11% in comparison with that of pure EP. This study is valuable for expanding the application of flame-retardant EP/DMP thermosets in emerging fields.

## 1. Introduction

EP is an important thermosetting plastic. Due to its excellent adhesion, mechanical performance and corrosion resistance, it is widely used in the manufacture of circuit boards, various adhesives and coatings [1,2,3,4]. However, EP is highly flammable and accompanied by large amounts of smoke toxic gases during its burning, which limits its application in most fields [5,6,7,8]. Meanwhile, with the increasing global attention to environmental protection and sustainability [9,10,11,12], it has become a general trend to make necessary flame retardant modifications to EP thermosets.

At present, the flame retardants used for EP were mainly phosphorus flame retardants, such as phosphate esters and phosphates [13,14,15]. Liang et al. [16] synthesized an aryl phosphinate dianhydride, BPAODOPE, which was used as a curing agent and flame retardant for the preparation of flame-retardant epoxy resins (EPs) by coupling it with another curing agent. The best performance of the flame-retardant specimen combination was achieved when the addition amount of BPAODOPE was 38% (phosphorus content of 1.75%), with an LOI value of 29.3, and passed the UL-94 V-0 rating. However, the high additive level resulted in a significant reduction in the mechanical properties of the EP thermosets, with the tensile and impact strengths reduced by 19% and 32%, respectively, compared to the pure EP. In addition, a novel P/N phosphate (NDP) was synthesized to prepare flame-retardant EP by You et al. [17]. The EP/NDP thermosets passed UL-94 V-0 rating when NDP was added at 11.4 wt%, but its flexural strength was reduced. In summary, the traditional phosphorous flame retardants added to EP often have low flame retardant efficiency and a significant impact on the mechanical performance of EP thermosets. In addition, with the miniaturization and integration of electronic and electrical appliances, EP thermosets needed to maintain its excellent comprehensive performance while improving its flame retardant performance [18,19,20]. Therefore, it was necessary to develop a novel phosphorous flame retardant to meet the high-performance flame-retardant EP.

According to previous studies [21,22,23,24], aryl phosphate flame retardants with similar EP structures had less effect on the mechanical performance of EP thermosets. Its flame retardant efficiency in EP was relatively low due to the presence of P-O groups alone, which needs to be further improved. As we all know, EP produces a large number of highly active free radicals during its decomposition process, and its efficient flame retardancy cannot be realized by catalytic charring alone [3,25]. Therefore, the efficient flame retardancy of EP thermosets can be realized while ensuring their excellent mechanical properties through the introduction of a large number of P-Ph groups into the traditional phosphate ester structure and the synergistic interaction with P-O groups [26,27].

In this study, the aryl phosphate flame-retardant DMP was successfully prepared and introduced into the EP matrix. The flame retardancy, combustion behavior, mechanical and thermal properties of EP/DMP thermosets were studied in detail. Meanwhile, the flame retardant mechanism of DMP in EP was also exposed.

## 2. Experimental

### 2.1. Materials

**Figure 1:**
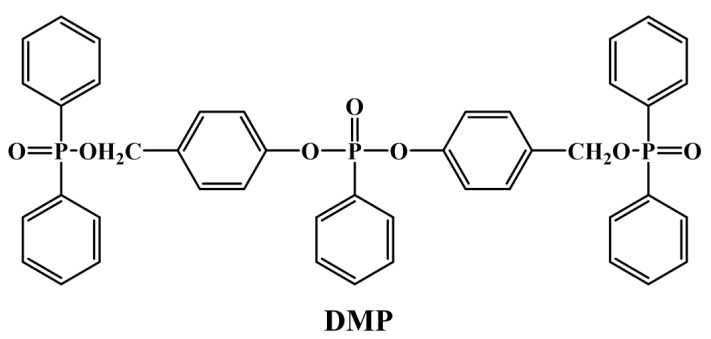
Chemical structure of flame-retardant DMP.

Bis(4-(((diphenylphosphoryl)oxy)methyl)phenyl)phenylphosphonate (DMP) was prepared in the laboratory (Figure 1) [28]. Metaphenylenediamine was purchased from Macklin Biochemical Technology Co., Ltd. (Shanghai, China), and epoxy resin (E-51, epoxy equivalent = 184 g/mol) was provided by Xingchen Synthetic Materials Co., Ltd. (Nantong, China).

### 2.2. Preparation of Flame-Retardant EP Thermosets

After DMP was melted at high temperature, EP and DMP were stirred at 80 °C for 5 min to ensure complete dissolution of DMP in the EP matrix. The curing agent m-phenylenediamine (MPA) was introduced to the EP/DMP and thoroughly mixed. The mixture was subjected to ultrasonic defoaming in a 60 °C for 1 min and injected into pre-prepared molds. The curing process was conducted at 105 °C for 1 h and 150 °C for 2 h. After cooling to room temperature, the EP/DMP composite was obtained, with its detailed composition shown in Table 1. The usage ratio of the curing agent MPA was calculated according to Equation (1):

(1) $$W_{a}=\frac{M\times E_{v}}{N\times 100}$$

where *W_a_* represents the percentage of the curing agent in EP, *M* is the molecular weight of m-phenylenediamine, *E_v_* is the epoxy equivalent, and *N* is the number of active hydrogen atoms.

### 2.3. Measurements

Limiting oxygen index (LOI) values were performed on a JF-3 oxygen index meter (Jiangning, China) according to ASTM D2863 standard and vertical burning (UL-94) tests were measured by a CZF-2-type instrument (Jiangning, China) according to ASTM D3801 standard. The combustion behavior was performed with a Fire Testing Technology cone calorimeter (West Sussex, UK) at a heat flux of 50 kW·m^−2^.

TG-IR of the samples was measured by TGA 8000 thermogravimetric analyzer and FTIR spectrophotometer (MA, USA). Laser Raman spectroscopy (LRS) was measured on a confocal Raman spectroscopic system (London, UK) by a 633 nm laser.

The mechanical performance was performed on regeer mechanical instrument according to ASTM D412 standards. Dynamic mechanical analysis (DMA) was characterized by a PE DMA8000 dynamic mechanical analyzer (MA, USA).

## 3. Results and Discussion

### 3.1. Fire Safety Analysis of EP/DMP Thermosets

**Figure 2:**
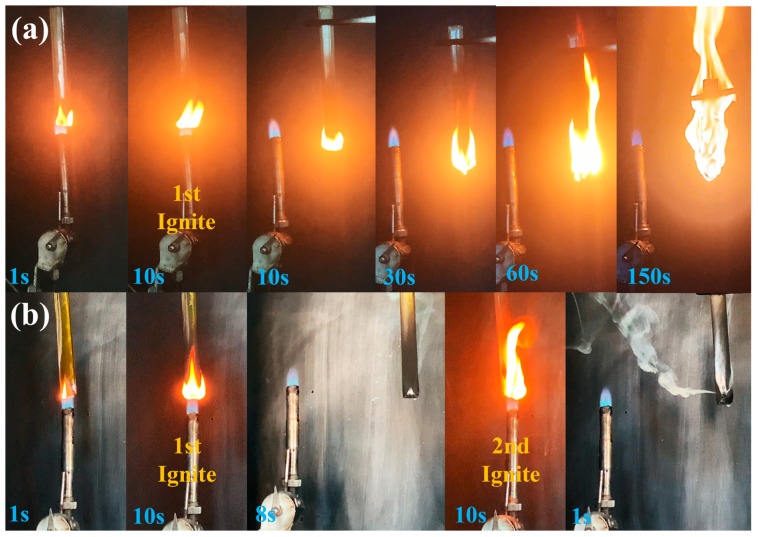
Screenshots of pure EP (**a**) and EP/9 wt%DMP (**b**) during vertical burning tests.

The DMP was introduced into the EP matrix and the flame retardancy of EP/DMP thermosets was studied using vertical combustion and LOI tests. During the UL-94 tests (Figure 2), pure EP ignited quickly upon initial combustion, and it was completely burned out at 150 s, indicating its flammability and underscoring the importance of flame retardant modifications for practical applications. As shown in Table 1, pure EP had no rating in the UL-94 test, and its limiting oxygen index (LOI) was only 24.5%. However, the flame retardancy of the EP/DMP thermosets was improved with the increase in DMP content. When 6 wt% DMP was added, the EP/6 wt% DMP thermosets passed a UL-94 V-2 rating with an LOI of 33.1%. EP/9 wt% DMP thermosets passed the UL-94 V-0 rating, and the LOI reached 35.0%, demonstrating its excellent flame retardant efficiency. Furthermore, the t_1_ + t_2_ times for the EP/DMP thermosets significantly decreased with the increase in DMP content. This was attributed to DMP catalyzing the dehydration and charring of EP and the dense char layer. Thus, EP/DMP thermosets obtained higher flame retardant efficiency.

### 3.2. Combustion Behavior and Thermal Property Analysis of EP/DMP Thermosets

**Figure 3:**
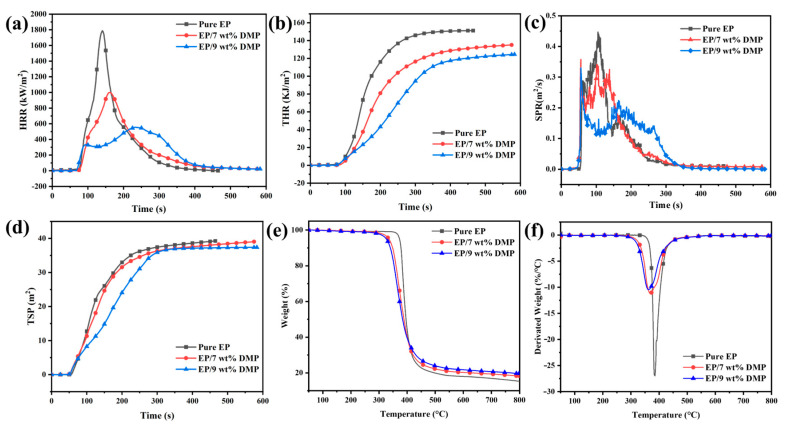
HRR (**a**), THR (**b**), SPR (**c**) and TSP (**d**) curves of E pure EP, EP/7 wt% DMP and EP/9 wt% DMP; TG (**e**) and DTG (**f**) curves of pure EP, EP/7 wt% DMP and EP/9 wt% DMP under nitrogen atmosphere.

The combustion behavior of EP/DMP thermosets was analyzed by simulating a real fire (Figure 3 and Table 2). The TTI of EP/7 wt% DMP and EP/9 wt% DMP thermosets decreased from 56 s of EP to 53 s and 52 s, indicating that DMP promoted the early degradation and charring of the EP matrix, which was conducive to the enhancement of the flame retardancy for thermosets.

Figure 3a,b show HRR and THR curves of pure EP and EP/DMP thermosets (Figure 3). Pure EP burned quickly after ignition and released a large amount of heat, and its PHRR was 1786.7 kW·m^−2^ at 86 s. The PHRR of EP/DMP was significantly decreased and its corresponding time was delayed, and the PHRR of EP/9 wt% DMP thermosets was 555.6 kW·m^−2^ at 180 s, which was 68.9% lower compared with pure EP. Meanwhile, the THR of EP/9 wt% DMP thermosets was reduced from 150.8 MJ·m^−2^ for EP to 123.5 MJ·m^−2^. This was attributed to DMP decomposing and catalyzing the dehydration of EP into charring, with the char layer exerting a shielding effect, restraining the further combustion degradation of the internal EP matrix.

In addition, large amounts of smoke release suffocate people in real fires. Therefore, there is a need to reduce the smoke released from the combustion of materials. Figure 3c,d show the TSP and SPR curves of pure EP and EP/DMP. The pattern of the SPR curves was similar to the HRR curves, and pure EP burned quickly, releasing a large amount of smoke as soon as it was ignited, producing a maximum smoke release of 0.45 m^−2^·s^−1^ at 105 s. The PSPR values of EP/7 wt% DMP and EP/9 wt% DMP were 0.30 and 0.22 m^−2^·s^−1^, which were 33.1% and 49.9% lower than the PSPR of pure EP. This was attributed to the catalytic charring of DMP and the shielding effect of the char layer decreasing the SPR of EP/DMP. Thus, the TSP of EP/7 wt% DMP and EP/9 wt% DMP also decreased from 39.3 m^2^ of EP to 39.0 and 37.4 m^2^, which indicated that the introduction of DMP improved the smoke suppression properties of EP thermosets along with their flame retardant properties.

The thermal degradation behavior of DMP and EP/DMP was studied using TG tests (Table 3 and Figure 3e,f). The T_initial_ (initial thermal decomposition temperature) of DMP was 283 °C, which can meet the curing temperature requirements of EP thermosets. Under nitrogen atmosphere, pure EP had one degradation stage and was distributed between 350 and 460 °C. This degradation stage was due to the breakage of the crosslinking network of EP. However, the initial decomposition temperatures of EP/7 wt% DMP and EP/9 wt% DMP thermosets were decreased from 349 °C of pure EP to 268 °C and 237 °C, indicating that the decomposition of DMP promoted the early cross-linking of the EP to form residual char. The formed char layer has a certain barrier effect. Thus, with the increase in DMP content, the maximum thermal degradation peak (R_peak_) of EP/DMP was significantly lower compared to pure EP (26.8%·min^−1^/°C), and the R_peak_ of EP/9 wt% DMP was only 10.4%·min^−1^/°C. Meanwhile, the char residues of EP/7 wt% DMP and EP/9 wt% DMP thermosets were enhanced from 15.4% of pure EP to 18.3% and 19.6% at 800 °C, consistent with the test results of CC tests.

### 3.3. Gas Phase Analysis of EP/DMP Thermosets

**Figure 4:**
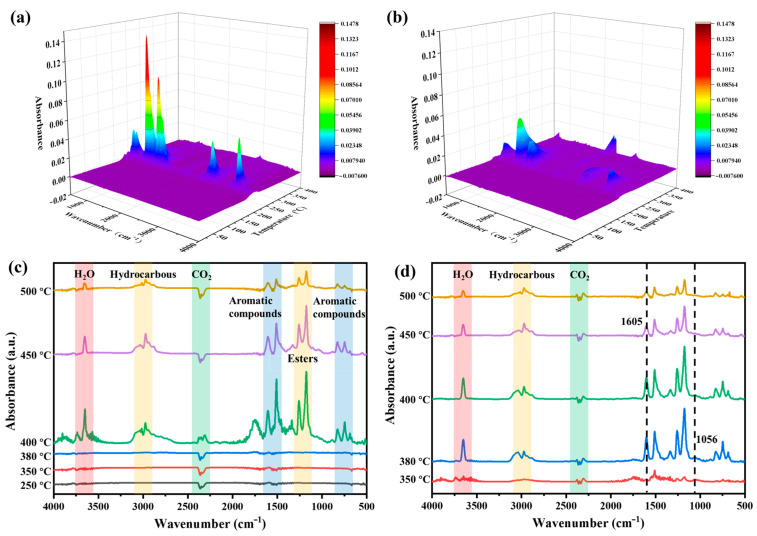
Three-dimensional TG-IR of pure EP (**a**) and EP/9 wt% DMP (**b**) thermosets; infrared images of pyrolysis products of pure EP (**c**) and EP/9 wt% DMP (**d**) thermosets at different temperatures.

In order to study the effect of DMP for EP in the gas phase, the pyrolysis products of EP and EP/DMP thermosets were analyzed using the TG-IR coupling technique [29,30]. The 3D TG-IR spectra of pure EP and EP/9 wt% DMP thermosets as well as the FTIR spectra of their pyrolysis products at different temperatures are shown in Figure 4. It is evident that the total amount of pyrolysis products of pure EP was higher than that of EP/9 wt% DMP thermosets, which matched with the reduction in smoke release mentioned above. It indicated that the introduction of DMP significantly decreased the concentration of combustion gases. As shown in Figure 4c, the main pyrolysis products of pure EP were H_2_O (3650 cm^−1^), hydrocarbons (2969 cm^−1^), CO_2_ (2347 cm^−1^), aromatic compounds (1450–1550 cm^−1^) and esters (1140–1280 cm^−1^) [31,32]. The main pyrolysis products of EP/9 wt% DMP thermosets were similar to pure EP (Figure 4d). However, a new characteristic peak of P-O appeared at 1056 cm^−1^, which demonstrated that DMP can produce phosphorus-containing free radicals during its decomposition process. In Figure 4c, the pyrolysis products of EP/9 wt% DMP composites already had an obvious peak at 380 °C, while pure EP had almost no decomposition products at this time. This indicated that DMP promoted the early degradation of the EP matrix. In addition, the aromatic compounds of EP/9 wt% DMP composites have relatively late and lower peaks, indicating that more thermal degradation products of EP/DMP composites were catalyzed into char, and the char layer effectively inhibited the escape of the subsequent decomposition gases. Therefore, the gas-phase combustibles of EP/DMP thermosets were obviously lower than that of pure EP.

### 3.4. Condensed Phase Analysis of EP/DMP Thermosets

**Figure 5:**
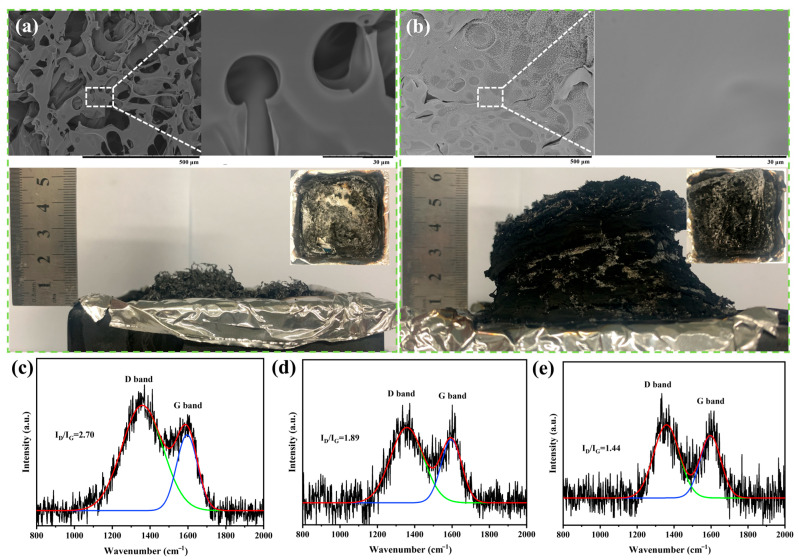
Digital and SEM images of the char residues for pure EP (**a**) and EP/9 wt% DMP (**b**); LRS curves of pure EP (**c**), EP/7 wt% DMP (**d**) and EP/9 wt% DMP (**e**) after CC tests.

The digital photographs and SEM images of the char residue of pure EP and EP/9 wt% DMP thermosets after cone calorimetry (CC) tests are shown in Figure 5a,b. Under the continuous thermal radiation, the char residue of EP was relatively small and discontinuous. The height of the residual char was only 1.8 cm, and there were many voids and unevenness in the microscopic structure of residual char. With the introduction of DMP, the amount of residual char for EP/DMP significantly improved, and the height of residual char reached 5.9 cm, which was 3.27 times that of pure EP. The surface of residual char for EP/DMP was dense and homogeneous, and the structure of the char layer was more stable and solid. This expanded char layer exerted an excellent barrier effect, effectively limiting the release of heat and flammable gases and protecting the internal EP matrix. Thus, DMP showed excellent flame retardant and smoke suppression properties in EP.

**Figure 6:**
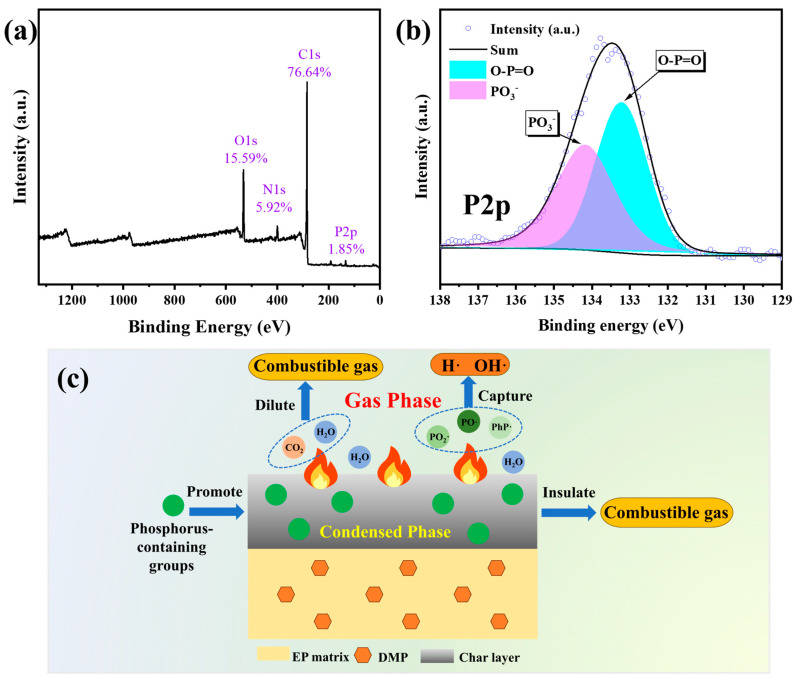
XPS analysis of char residue in EP/9 wt% DMP thermosets after CC tests: XPS full scan-survey spectra (**a**); high-resolution XPS spectra of P2p (**b**); schematic diagram of flame retardant mechanism of EP/DMP thermosets (**c**).

LRS and XPS analysis of the char layer for pure EP and EP/DMP after CC tests are shown in Figure 5c–e and Figure 6. The I_D_/I_G_ value of the char layer for pure EP was 2.70, and the I_D_/I_G_ values of the char layers of EP/7 wt% DMP and EP/9 wt% DMP were decreased to 1.89 and 1.44 compared with that of pure EP. This indicated that the introduction of DMP improved the partial graphitization degree of EP, which gave the char layer of EP/DMP high thermal stability and strength. In Table 4 and Figure 6, a new P element was detected in the residual char of EP/DMP, and the C content was obviously improved compared with pure EP. The P2p peaks at 133.20 and 134.20 eV were attributed to PO_3_ and O=P-O structures [33,34]. This demonstrated that the phosphoric acid and pyrophosphate from the decomposition of DMP promoted the cross-linking and charring of residual char, and the dense, uniform, and expanded char layer effectively exerted a shielding effect in the condensed phase. Therefore, EP/DMP achieved excellent fire safety.

Based on the above experimental results, the flame retardant mechanism of EP/DMP is proposed in Figure 6a-c. In gas phase, non-combustible gases such as CO_2_ and H_2_O from the early thermal decomposition of EP/DMP diluted the concentration of the combustible gas, and the P·, Ph· and PO· from the decomposition of DMP can capture H·, O· and HO·, thereby interrupting the combustion chain reaction. The phosphoric acid compounds produced by the degradation of DMP promoted the dehydration and cross-linking of EP to form a dense and uniform residual char in the condensed phase. Meanwhile, the escape of a large amount of non-combustible gas in the gas phase caused the char layer to expand. The expanded and compact char layer isolated the combustible gas, reduced the heat transfer rate, and protected the internal EP matrix from further degradation. Thus, EP/DMP exhibited excellent flame retardancy.

### 3.5. Mechanical Performance of EP/DMP Thermosets

**Figure 7:**
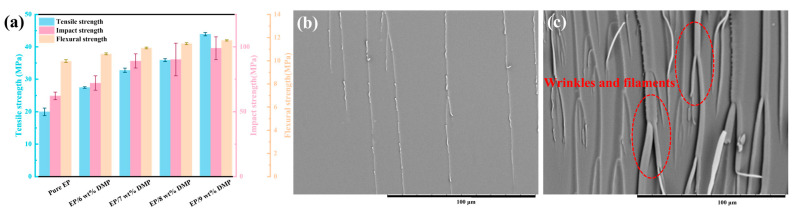
Tensile strength, impact strength and flexural strength of pure EP and EP/DMP thermosets (**a**); SEM images of fracture-section for pure EP (**b**) and EP/9 wt% DMP (**c**) thermosets after impact tests.

The mechanical performance of EP/DMP was characterized (Figure 7a). The tensile strength, flexural strength and impact strength of pure EP were 19.97, 89.06 and 6.96 MPa. It is evident that the tensile, flexural and impact strength of EP/9 wt% DMP was improved with the introduction of DMP. The tensile strength, flexural strength and impact strength of the EP/DMP increased to 43.92 MPa, 104.96 MPa and 11.09 MPa, which were 119.93%, 17.85% and 59.34% higher than those of pure EP. This was attributed to the polyaromatic structure of DMP, which provides high rigidity, and the conjugation of DMP with EP, which increased the energy required for the fracture of EP/DMP thermosets. Meanwhile, DMP had good compatibility with the EP matrix and limited the friction between EP molecular chains, thus improving the mechanical performance of EP/DMP thermosets. EP/DMP were in sharp contrast to those of traditional phosphorus-based flame-retardant EP, resulting in a reduction in the mechanical performance of EP thermosets.

To further study the effect of DMP on the compatibility and mechanical performance of EP, the impact section was analyzed using SEM. The cross section of pure EP was smooth with a single crack extending in a straight line and clearly exhibited brittle fracture. However, the impact cross section of the EP/9 wt% DMP thermosets was rougher and had obvious wrinkles and filaments. The filaments showed a good energy absorption effect [35,36,37,38,39], and the plastic deformation of the EP matrix promoted the dissipation of fracture energy and the formation of wrinkled morphology, as reflected in the improvement of impact toughness [40,41]. The introduction of DMP endowed the EP/DMP thermosets with excellent tensile, bending and impact properties, solving the problem of poor compatibility and mechanical properties of traditional phosphorus-based flame retardants in EP.

**Figure 8:**
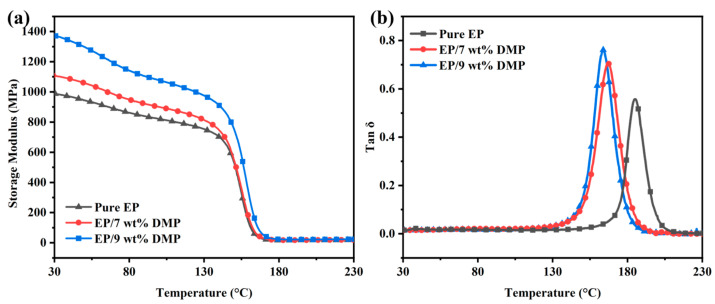
Temperature-dependent curves of storage modulus (**a**) and Tanδ (**b**) of pure EP, EP/7 wt% DMP and EP/9 wt% DMP thermosets.

Then, the dynamic mechanical analyzer (DMA) was used to analyze the mechanical relaxation and molecular motion of the EP/DMP thermosets as a function of temperature, thereby characterizing the blending compatibility of the thermosets. Figure 8 showed the storage modulus and loss tangent curves of pure EP, EP/7 wt% DMP, and EP/9 wt% DMP thermosets as a function of temperature. With the introduction of DMP, the storage modulus of EP/DMP thermosets was also improved. At 30 °C, the storage modulus of EP/7 wt% DMP and EP/9 wt% DMP increased from 987.1 MPa of pure EP to 1104.8 MPa and 1370.9 MPa (Table 5). This was due to the high strength and stiffness of the DMP containing polyaromatic structures, which acted as physical cross-linking points in the EP matrix. In addition, the aromatic ring structure in DMP exerted the conjugate stacking effect in EP and improved the storage modulus of the EP/DMP thermosets. In Figure 8b, both EP/DMP and EP have only one glass transition temperature (T_g_), and the T_g_ of EP/DMP decreased with the introduction of DMP. This was attributed to the DMP having good compatibility with the EP matrix and melting into a liquid state at high temperature, thus reducing the movement resistance between molecular chains and providing a certain lubrication effect for the movement of chain segments. However, when the peak position of Tan δ moved towards the low-temperature position, the molecular chain segments could absorb more energy during the movement, thus exhibiting better toughness. As a result, the toughness of EP/DMP thermosets was further improved.

## 4. Conclusions

In this paper, a novel arylphosphate flame retardant, bis(4-(((diphenylphosphoryl)oxy)methyl)phenyl)phenylphosphonate (DMP), was successfully synthesized. It was introduced into EP to prepare flame-retardant EP/DMP thermosets. When the addition amount of DMP was 9 wt%, the EP/9 wt% DMP passed the UL-94 V-0 rating, and its LOI increased from 24.5% of pure EP to 35.0%. The phosphorus-containing radicals from the decomposition of DMP effectively captured H·, O· and HO· during the decomposition of EP. Moreover, the phosphoric compounds from DMP decomposition promoted the degradation and charring of the EP matrix, and the dense and compact char residue decreased the release of heat and smoke. Therefore, compared with pure EP, the PHRR, THR, PSPR and TSP of the EP/9 wt% DMP thermosets were decreased by 68.9%, 18.1%, 51.1% and 4.8%, respectively. Meanwhile, the mechanical performance and storage modulus of EP/DMP thermosets were clearly improved due to the excellent compatibility and intermolecular forces between the DMP and EP matrix, and the tensile strength, flexural strength and impact strength of EP/9 wt% DMP were enhanced by 116%, 17.8% and 58.6% compared with pure EP. To sum up, EP/DMP thermosets have excellent flame retardant and mechanical properties and provided a new strategy for the high-performance EP thermosets.

## Figures and Tables

**Table 1:** UL-94 and LOI test results of EP/DMP thermosets.

Sample	P Content (wt%)	t_1_ + t_2_(s)	Dripping	UL-94	LOI (%)
Pure EP	0	>60	No	Fail	24.5
EP/6 wt%DMP	0.72	41.32	No	V-2	33.1
EP/7 wt%DMP	0.84	18.22	No	V-1	34.4
EP/8 wt%DMP	0.96	12.45	No	V-1	34.7
EP/9 wt%DMP	1.08	8.78	No	V-0	35.0

**Table 2:** Cone calorimetry data of EP and EP/DMP composites.

Properties	Pure EP	EP/7 wt% DMP	EP/9 wt% DMP
TTI (s)	56	53	52
Peak-HRR (kW·m^−2^)	1786.7	998.0	555.7
t_Peak-HRR_ (s)	86	110	180
THR (MJ·m^−2^)	150.8	134.3	123.5
Peak-SPR (m^−2^·s^−1^)	0.45	0.32	0.22
t_Peak-SPR_ (s)	105	143	164
TSP (m^2^)	39.3	39.0	37.4
COY (kg·kg^−1^)	0.19	0.30	0.31
Peak-COPR (g·s^−1^)	4.5	2.0	1.9
t_Peak-COPR_ (s)	202	259	289
CO_2_Y (kg·kg^−1^)	9.7	2.0	3.9
Peak-CO_2_PR (g·s^−1^)	331.6	82.6	60.0
Residual mass (%)	6.5	10.3	15.2

**Table 3:** Thermal degradation analysis data of pure EP, EP/7 wt% DMP and EP/9 wt% DMP under nitrogen atmosphere.

Sample	T_initial_ (°C)	R_peak1_/T_peak1_ (%^.^min^−1^/°C)	Char Residue (wt%)
			800 °C
Pure TPU	349	26.8/385	15.4
EP/7 wt% DMP	268	11.2/369	18.3
EP/9 wt% DMP	237	10.4/363	19.6

**Table 4:** Element relative content of the char residue for EP thermosets.

Element	Pure EP		EP/9 wt% DMP	
	Binding Energy (eV)	Element Content (%)	Binding Energy (eV)	Element Content (%)
C	284.51	73.04	284.08	76.64
O	532.08	12.63	532.08	15.59
N	400.08	14.33	400.08	5.92
P	—	—	133.09	1.85

**Table 5:** DMA data of EP and EP/DMP thermosets.

Sample	T_g_ (°C)	Storage Modulus at 30 °C (MPa)	Tan δ
Pure EP	185.7	987.1	0.557
EP/7 wt%DMP	167.1	1104.8	0.706
EP/9 wt%DMP	163.7	1370.9	0.761

## Data Availability

The original contributions presented in the study are included in the article, further inquiries can be directed to the corresponding authors.

## References

[1] Zhi M., Yang X., Fan R., Yue S., Zheng L., Liu Q., He Y. (2022). A comprehensive review of reactive flame-retardant epoxy resin: Fundamentals, recent developments, and perspectives. Polym. Degrad. Stab..

[2] Yao Z., Reinmöller M., Ortuño N., Zhou H., Jin M., Liu J., Luque R. (2023). Thermochemical conversion of waste printed circuit boards: Thermal behavior, reaction kinetics, pollutant evolution and corresponding controlling strategies. Prog. Energy Combust. Sci..

[3] Jin F.-L., Li X., Park S.-J. (2015). Synthesis and application of epoxy resins: A review. J. Ind. Eng. Chem..

[4] Gu H., Ma C., Gu J., Guo J., Yan X., Huang J., Zhang Q., Guo Z. (2016). An overview of multifunctional epoxy nanocomposites. J. Mater. Chem. C.

[5] Zhang J., Mi X., Chen S., Xu Z., Zhang D., Miao M., Wang J. (2020). A bio-based hyperbranched flame retardant for epoxy resins. Chem. Eng. J..

[6] Chruściel J.J., Leśniak E. (2015). Modification of epoxy resins with functional silanes, polysiloxanes, silsesquioxanes, silica and silicates. Prog. Polym. Sci..

[7] Yang Q., Wang J., Chen X., Yang S., Huo S., Chen Q., Guo P., Wang X., Liu F., Chen W. (2023). A phosphorus-containing tertiary amine hardener enabled flame retardant, heat resistant and mechanically strong yet tough epoxy resins. Chem. Eng. J..

[8] Bifulco A., Varganici C., Rosu L., Mustata F., Rosu D., Gaan S. (2022). Recent advances in flame retardant epoxy systems containing non-reactive DOPO based phosphorus additives. Polym. Degrad. Stab..

[9] Sauceda-Oloño P.Y., Dona N.L.K., Smith R.C. (2024). The quest for environmentally benign plastics: Advances in greener and more sustainable flame retardant formulations. Green Chem. Lett. Rev..

[10] Qi Y., Weng Z., Kou Y., Song L., Li J., Wang J., Zhang S., Liu C., Jian X. (2020). Synthesize and introduce bio-based aromatic s-triazine in epoxy resin: Enabling extremely high thermal stability, mechanical properties, and flame retardancy to achieve high-performance sustainable polymers. Chem. Eng. J..

[11] Yang H., Yu B., Xu X., Bourbigot S., Wang H., Song P. (2020). Lignin-derived bio-based flame retardants toward high-performance sustainable polymeric materials. Green Chem..

[12] Zhou S., Tao R., Dai P., Luo Z., He M. (2020). Two-step fabrication of lignin-based flame retardant for enhancing the thermal and fire retardancy properties of epoxy resin composites. Polym. Compos..

[13] Huo S., Song P., Yu B., Ran S., Chevali V.S., Liu L., Fang Z., Wang H. (2021). Phosphorus-containing flame retardant epoxy composites: Recent advances and future perspectives. Prog. Polym. Sci..

[14] Velencoso M.M., Battig A., Markwart J.C., Schartel B., Wurm F.R. (2018). Molecular Firefighting—How Modern Phosphorus Chemistry Can Help Solve the Challenge of Flame Retardancy. Angew. Chem. Int. Ed..

[15] Cuminet F., Vanachte N., Farina C., Denis M., Negrell C., Caillol S., Dantras É., Leclerc É., Ladmiral V. (2024). Phosphorus acid: An asset for flame-retardant sustainable vitrimers. Polym. Chem..

[16] Liang B., Cao J., Hong X., Wang C. (2013). Synthesis and properties of a novel phosphorous-containing flame-retardant hardener for epoxy resin. J. Appl. Polym. Sci..

[17] You G.Y., Cheng Z.Q., Peng H., He H. (2015). Synthesis and performance of a novel nitrogen-containing cyclic phosphate for intu-mescent flame retardant and its application in epoxy resin. J. Appl. Polym..

[18] Fu Z., Ma Z., Liu J., Li C., Liu C., Wang Q., Song L., Yu Q., Cheng G., Han Y. (2024). Phosphorus-containing active esters modified dicyclopentadiene epoxy resins with simultaneously improved flame retardancy, thermal stability, and dielectric properties. Chem. Eng. J..

[19] Lou S., Wang S., Zhang L., Liu J., Tang T. (2024). Intrinsic flame-retardant epoxy resin based on phosphorus-containing liquid imidazole compound: Simultaneously improving fire safety, mechanical property and heat resistance. Chem. Eng. J..

[20] Hamciuc C., Vlad-bubulac T., Serbezeanu D., Lisa G., Anghel I., Preda D.M. (2023). Eco-friendly flame retardant epoxy nanocomposites based on polyphos-phonate and halloysite nanotubes. J. Vinyl. Addit. Techn..

[21] Hupp V., Schartel B., Flothmeier K., Hartwig A. (2024). Pyrolysis and flammability of phosphorus based flame retardant pressure sensi-tive adhesives and adhesive tapes. J. Anal. Appl. Pyrol..

[22] Bereska A., Kafarski P., Bereska B., Tkacz B., Iłowska J., Lenża J. (2017). The Application of Organophosphorus Flame-Retardants in Epoxy Resin. J. Vinyl Addit. Technol..

[23] Guo Z., Wang C., Li J. (2016). An Intumescent-Like Flame-Retardant Effect of Hollow Carbon Precursor on Acryloni-trile-Butadiene-Styrene/Oligomeric Aryl Phosphate/Novolac Epoxy Composites. Polym-Plast. Technol..

[24] Bekeshev A., Mostovoy A., Shcherbakov A., Zhumabekova A., Serikbayeva G., Vikulova M., Svitkina V. (2023). Effect of phosphorus and chlorine containing plasticizers on the physicochemical and mechanical properties of epoxy composites. J. Compos. Sci..

[25] Ma L., Wang Y., Gao J., Zhu Z. (2024). Construction of MXene-based flame retardant with multiple carbonization towards for reducing fire hazard and smoke release of epoxy resin. Eur. Polym. J..

[26] Yu M., Chu Y., Xie W., Fang L., Zhang O., Ren M., Sun J. (2024). Phosphorus-containing reactive compounds to prepare fire-resistant vinyl resin for composites: Effects of flame retardant structures on properties and mechanisms. Chem. Eng. J..

[27] Tao Z., Yong L. (2023). Preparation of high-transparency phosphenanthrene-based flame retardants and studies of their flame-retardant properties. Polymers.

[28] Zhang W., Xu Y., Yan C., Gang Y., Qin A., Xu K., Xu M., Li B., Liu L. (2024). Preparation of novel and efficient arylphosphonate flame retardants for simultaneously en-hancement of fire safety and UV-shielding properties of transparent thermoplastic polyurethane. Polym. Degrad. Stabil..

[29] Bai Z., Wang X., Tang G., Song L., Hu Y., Yuen R.K. (2013). Structure–property relationships of synthetic organophosphorus flame retardant oligomers by thermal analysis. Thermochim. Acta.

[30] Wang D., Mu X., Cai W., Song L., Ma C., Hu Y. (2018). Constructing phosphorus, nitrogen, silicon-co-contained boron nitride nanosheets to rein-force flame retardant properties of unsaturated polyester resin. Compos. Part A Appl. Sci. Manuf..

[31] Xu Y., Yan C., Du C., Xu K., Li Y., Xu M., Bourbigot S., Fontaine G., Li B., Liu L. (2023). High-strength, thermal-insulating, fire-safe bio-based organic lightweight aerogel based on 3D network construction of natural tubular fibers. Compos. Part B Eng..

[32] Shi Y., Xu Y., Xu K., Yan C., Qin A., Du C., Xu M., Wang C., Li B., Liu L. (2024). Fire-resistant and thermal-insulating alginate aerogel with intelligent bionic armor for exceptional mechanical and fire early-warning performance. Chem. Eng. J..

[33] Zhu P., Feng L., Liu J., Wang M., Ma N., Tsai F.-C., Tang Y. (2024). Influence of concentration, dispersibility, compatibility and orientation of rod-like cellulose nanocrystals in epoxy resin on the mechanical performance of their composite films. Prog. Org. Coatings.

[34] Wang J., Yu X., Si J., Shao X., Zhao S., Ding G. (2024). Tailoring compatibility and mechanical properties of cold-mixed epoxy asphalt via external epoxy group content manipulation. Constr. Build. Mater..

[35] Vincent V.A., Kailasanathan C., Shanmuganathan V.K., Kumar J.V.S.P., Arun Prakash V.R. (2022). Strength characterization of caryota urens fibre and aluminium 2024-T3 foil multi-stacking sequenced SiC-toughened epoxy structural composite. Biomass Convers. Biorefinery.

[36] Liu X.-F., Xiao Y.-F., Luo X., Liu B.-W., Guo D.-M., Chen L., Wang Y.-Z. (2022). Flame-Retardant multifunctional epoxy resin with high performances. Chem. Eng. J..

[37] Xu Y., Qiu Y., Yan C.T., Liu L.B., Xu M.J., Li B. (2021). A novel and multifunctional flame retardant nucleating agent towards superior fire safety and crystallization properties for biodegradable poly (lactic acid). Adv. Powder Technol..

[38] Yang K., Long Y., Luo J., Zhang S., Feng W., Tian W., Yan H. (2024). Bridging solvent-free polyamic acid and epoxy resin by Si-O-C hyperbranched polymer for enhanced compatibility, toughness and self-lubrication performance. Chem. Eng. J..

[39] Liu L., Xu Y., Pan Y., Xu M., Di Y., Li B. (2021). Facile synthesis of an efficient phosphonamide flame retardant for simultaneous enhancement of fire safety and crystallization rate of poly (lactic acid). Chem. Eng. J..

[40] Mi X., Liang N., Xu H., Wu J., Jiang Y., Nie B., Zhang D. (2022). Toughness and its mechanisms in epoxy resins. Prog. Mater. Sci..

[41] Teng N., Dai J., Wang S., Hu J., Liu X. (2022). Hyperbranched flame retardant for epoxy resin modification: Simultaneously improved flame retardancy, toughness and strength as well as glass transition temperature. Chem. Eng. J..

